# Portable Automated Oxygen Administration System for hypoxaemic patients

**DOI:** 10.1186/s40064-016-2102-z

**Published:** 2016-04-18

**Authors:** Khawla Alzoubi, Ziyad Alguraan, Omar M. Ramahi

**Affiliations:** Department of Electrical Engineering, College of Engineering, Qatar University, Doha, Qatar; ECE Department, University of Waterloo, Waterloo, Canada; RAlsalam Medical Group, Riyadh, 11433 Saudi Arabia

**Keywords:** Hypoxaemia, Oxygen therapy, Intelligent oxygen delivery, Hypoxaemic patient, Oxygen administration

## Abstract

Oxygen is a lifesaving medication that should be offered with an administration to a patient who suffers from oxygen deficiency to avoid toxic effects of excessive oxygen supplement as well as to minimize the exposure to hypoxaemia. This work aims to automate the process of administering oxygen delivery in order to extend the continuous oxygen administration process beyond the IC units, reduce the cost of oxygen administration in terms of well-trained health care providers and equipment, prolong the lifetime of oxygen supplement, and help in the process of weaning patient from oxygen. In this work, a prototype model for a Portable Automated Oxygen Delivery System that consists of two subsystems: an Oxygen Reader Subsystem and an Automated Adjustment Oxygen Delivery Subsystem, both communicating wirelessly, has been developed. The system promises significant benefits in improving the life quality of hypoxaemic patients as well as healthcare service for oxygen delivery administration.

## Background

Hypoxaemia, an oxygen deficiency in human blood, is a common symptom in many serious illnesses that mostly relate to the heart and lung (Pierson 2000). The prevalence of hypoxaemia in lung diseases is significant. According to European lung foundation, it is expected that in 2020, out of 68 million deaths worldwide, 11.9 million will be caused by lung diseases (Siniscalco et al. 2008). Every year an estimated 156 million new cases of pneumonia occur and 2 million children under the age of five years die from pneumonia because lack of proper treatment. According to World Health Organization (WHO), the median prevalence of hypoxaemia in pneumonia was 13 %, but the prevalence varied in different region. This corresponds to 15–27 million cases of hypoxaemic pneumonia per year (Subhi et al. 2009). In 2010, 300 million people were affected by asthma worldwide. In 2009 asthma caused 250,000 deaths globally (Brunner 2010). In a study that was conducted in India, 26 % of 51 children presenting to an emergency department in India with asthma had hypoxaemia (Moore and Pascual 2010). Most of the 250,000 deaths from asthma each year can be attributed to lack of proper treatment.

Chronic obstructive pulmonary disease (COPD) affects an estimated 210 million people worldwide (GARD 2014). It is the fourth most common cause of death in the United States, and it is expected that it will be the third leading cause of death in the world by 2030 (Abegnoli 2014). Over 80 % of the patients with advanced disease enrolled in the National Emphysema Treatment Trial were using some form of oxygen therapy (Kent et al. 2011). 90 % of COPD deaths were estimated to occur in low and middle income countries due to lack of proper long term oxygen treatment (European COPD Coalition 2015). Clearly, millions of cases of lung diseases are admitted to the healthcare facilities, causing millions of hypoxaemic patients to be under the risk of death and disability due to lack of proper oxygen treatment.

Hypoxaemia is strongly associated with in-patient death and disability (Duke et al. 2010). This association is more dominant in underserved populations due to the high cost associated with oxygen therapy, and overall deficiency in well-trained healthcare providers. Conventionally, oxygen therapy is commonly administered in intensive care units (ICUs) using a desktop Pulse-Oximeter by which healthcare providers continuously monitor patient’s oxygen and manually adjust the amount of supplemental oxygen to the hypoxaemic patient. ICUs are expensive in terms of equipment and healthcare delivery. Failure to admit patients with severe hypoxaemia to ICU due to bed capacity or misdiagnosis could lead to death or cause permanent disability. Furthermore, many studies have proven that excessive oxygen supplement has toxic effects (Crapo 1986; Fisher 1980; Jackson 1985).

To help more hypoxaemic patients to have administrated oxygen therapy in cost effective way, to reduce toxic effects of oxygen therapy, and to accelerate the process of oxygen weaning, a portable automated intelligent system that continuously monitors and adjusts oxygen delivery is needed. Recently, few studies have addressed the process of automated or computerized oxygen delivery process. Investigation and testing the feasibility of using an automated system for oxygen delivery was reported in Behbahani and Ali (2012), Lellouche and LHer (2012), Cirio and Nava (2011). These studies were based on using a commercial Pulse Oximeter and a developed automated computerized system that adjusts the oxygen flow based on a Pulse-Oximetry signal. In this work, we designed the Oxygen Reader Subsystem instead of using a commercial one for the following reasons: (1) To have control over processing the raw data with the aim to develop more robust algorithms that can provide higher reading accuracy for patients oxygen saturation and heart rate, even with patients movements and other external artifacts. (2) To customize our Oxygen Reader Subsystem to a wearable earlobe Pulse Oximeter with higher level of comfort and ease of use. (3) To implement Bluetooth technology in our Pulse Oximeter (Oxygen Reader Subsystem) to communicate with the Automated Oxygen Delivery Subsystem that administers the oxygen delivery based on patient needs. (4) To own the technology. Thus, we can control and reduce the overall cost of developing the Portable Automated Oxygen Delivery Subsystem, which in turn helps to reduce its price for commercialized. This in turn will help more hypoxemic patients, specifically in developing countries, to be able to acquire the entire system with reasonable price. (5) To have more control in reducing the overall power consumption by applying power management techniques which will prolong the battery life for our whole system as power consumption is a critical design issues in portable systems.

Generally speaking, all the previous works have shown promising results as demonstrated through clinical trials in terms of minimizing time of the exposure to hypoxaemia, reducing the chance of toxic effect of oxygen therapy, and prolong the life of oxygen supply where the oxygen supply is limited.

This paper describes the development of a Portable Automated Oxygen Administration System (PAOAS) which has two subsystems that communicate wirelessly: an Oxygen Reader Subsystem that measures the oxygen saturation level in human’s blood non-invasively, and an Automated Adjustment Oxygen Delivery Subsystem that adjusts the amount of supplemental oxygen for hypoxaemic patient based on his/her oxygen’s needs. To the best of our knowledge, this system will be the first system of its kind to be used in healthcare service to administer the process of oxygen therapy at hospital settings, pre-clinics, and home. Furthermore, as this system is portable and small, hypoxaemic patient can use it continuously during all life activities.

## Medical background

Oxygen is necessary for metabolic processes in human cells and tissues. Oxygen is delivered to human’s cells through the blood. Most of the oxygen (97 %) that is transported to blood from the lung’s alveolus is combined with the haemoglobin and (3 %) is dissolved in the plasma (Bukwirwa 1999a). Hypoxaemia is oxygen deficiency in arterial blood circulation (Eckman 2010). Oxygen deficiency in blood can lead to hypoxia, which is oxygen deficiency in tissues and cells. Tissues and cells use oxygen to perform essential life metabolic processes. Hypoxia affects the functionalities of cells, tissues, and organs. In severe cases, if not treated effectively in a timely manner, hypoxia could cause permanent disability or death within few minutes.

**Fig. 1:**
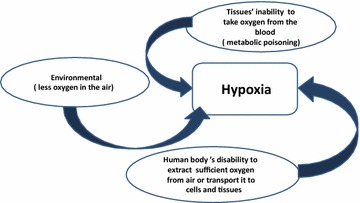
Main causes of hypoxia

**Fig. 2:**
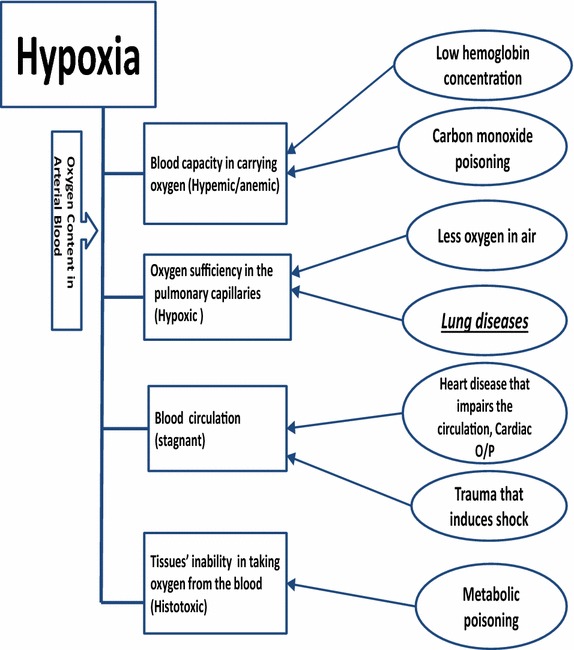
Hypoxia types

Generally, hypoxia could occur as a consequence to one of the following causes (see Fig. 1): Tissues’ inability to take oxygen from the blood (metabolic poisoning) (Liesivuori and Savolainen 1991), environmental conditions (less oxygen in the air) (Schoene 2001), or the human body’s disability to extract sufficient oxygen from air or transport it to cells and tissues (Rao et al. 2012). Medically, hypoxia can be classified into four types based on its causes as shown in Fig. 2. The oxygen content in arterial blood is affected by blood capacity in carrying oxygen and oxygen sufficiency in the pulmonary capillaries. The oxygen content of arterial blood ${\text {CaO}}_{2}$ is described using Eq. (1) (Bukwirwa 1999b), where Hb is the haemoglobin concentration (g/l), ${\text {SaO}}_{2}$ is the arterial Hb oxygen saturation, and ${\text {PaO}}_{2}$ is arterial oxygen partial pressure.

1 $${\text {CaO}}_{2}=(k1 \times {\text {Hb}} \times {\text {SaO}}_{2}+(K_{2} \times {\text {PaO}}_{2})$$

Oxygen delivery is affected by blood capacity in carrying oxygen, oxygen sufficiency in the pulmonary capillaries, and blood circulation. Global oxygen delivery is the amount of oxygen delivered to the whole body from the lungs. It is the product of total blood flow or cardiac output (CO) and the oxygen content of arterial blood ( ${\text {CaO}}_{2}$ ) and is usually expressed in ml/min (Bukwirwa 1999b).

2 $${\text {DO}}_{2}={\text {CO}} \times {\text {CaO}}_{2}$$

### Hypoxaemia detection and measuring

**Fig. 3:**
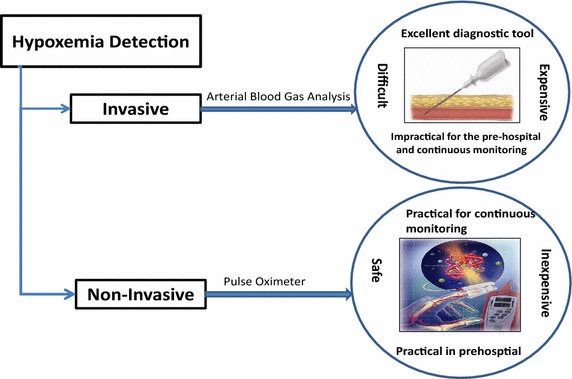
Hypoxaemia detection and measuring methods

To detect and measure the oxygen deficiency in human blood, invasive and non-invasive methods can be used as shown in Fig. 3. The invasive method requires a blood sample to be taken from patient, so, it is intrusive and impractical for continuous on-line monitoring, pre-hospital settings and home. To obtain the oxygen saturation in the blood (${\text {SaO}}_{2}$) invasively (Naeije and Barberá 2001), blood gas analysis technique is used. The blood gas analysis is costly in terms of equipment, storage, and healthcare providers.

On the other hand, the non-invasive method is safe, inexpensive and practical for continuous monitoring and pre-hospital settings. Pulse Oximeter was first introduced in 1980 to monitor the ${\text {SpO}}_{2}$ in the blood non-invasively. In this device, the oxygen saturation of the capillary blood $({\text {SpO}}_{2})$ is measured using radiation sensing techniques (Naeije and Barberá 2001).

### Oxygen therapy administration

Oxygen is a lifesaving medication that should be offered to hypoxaemic patient with administration to provide the patient with his/her oxygen needs as well as to avoid toxic effects of excessive oxygen supplement. Administered oxygen therapy requires monitoring patient’s oxygen level as well as adjusting the amount of oxygen delivery. Up to this date, this process of adjusting the supplemental oxygen is conducted manually. Furthermore, the process of administering oxygen therapy continuously is just performed in ICU and hospital settings by healthcare providers who make their decisions by monitoring the patient oxygen saturation using desktop Pulse Oximeter or blood gas analysis.

## Portable Automated Oxygen Administration System (PAOAS)

To avoid improper treatment of hypoxaemic patient and overcome the limitations of administering oxygen therapy, in this work, we report on our development of a prototype model for a Portable Automated Oxygen Administration System (PAOAS).

**Fig. 4:**
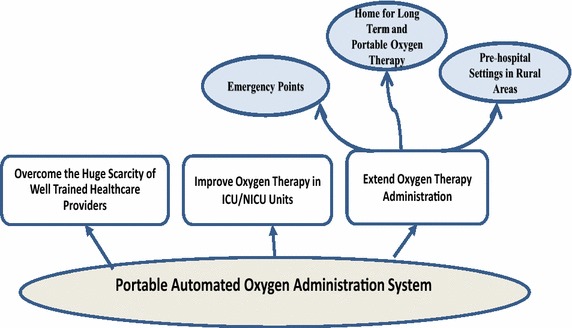
Promising benefits of PAOAS on healthcare services

**Fig. 5:**
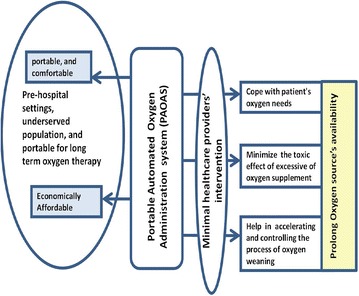
Design goals of PAOAS

The promising benefits of using the developed PAOAS for oxygen delivery administration to healthcare service are demonstrated by (see Fig. 4): (1) Improving the administered oxygen therapy in ICUs and hospitals settings, (2) Extending the administered oxygen therapy beyond the hospital settings, and (3) Overcoming the scarcity of well-trained healthcare providers. To achieve the intended benefits of the PAOAS, the design goals of such a system are depicted in Fig. 5.

In this work, the developed prototype model is capable of measuring the patient oxygen saturation non-invasively and adjusting the supplemental oxygen accordingly. In reality, providing the hypoxaemic patient by just the required amount of oxygen in a timely manner will minimize and avoid the toxic effects of oxygen therapy by continuously adjusting the supplemental oxygen based on his/her needs. This also will help the hypoxaemic patient to get the required amount of oxygen while carrying out his daily activities.

### Description of the Portable Automated Oxygen Administering System

**Fig. 6:**
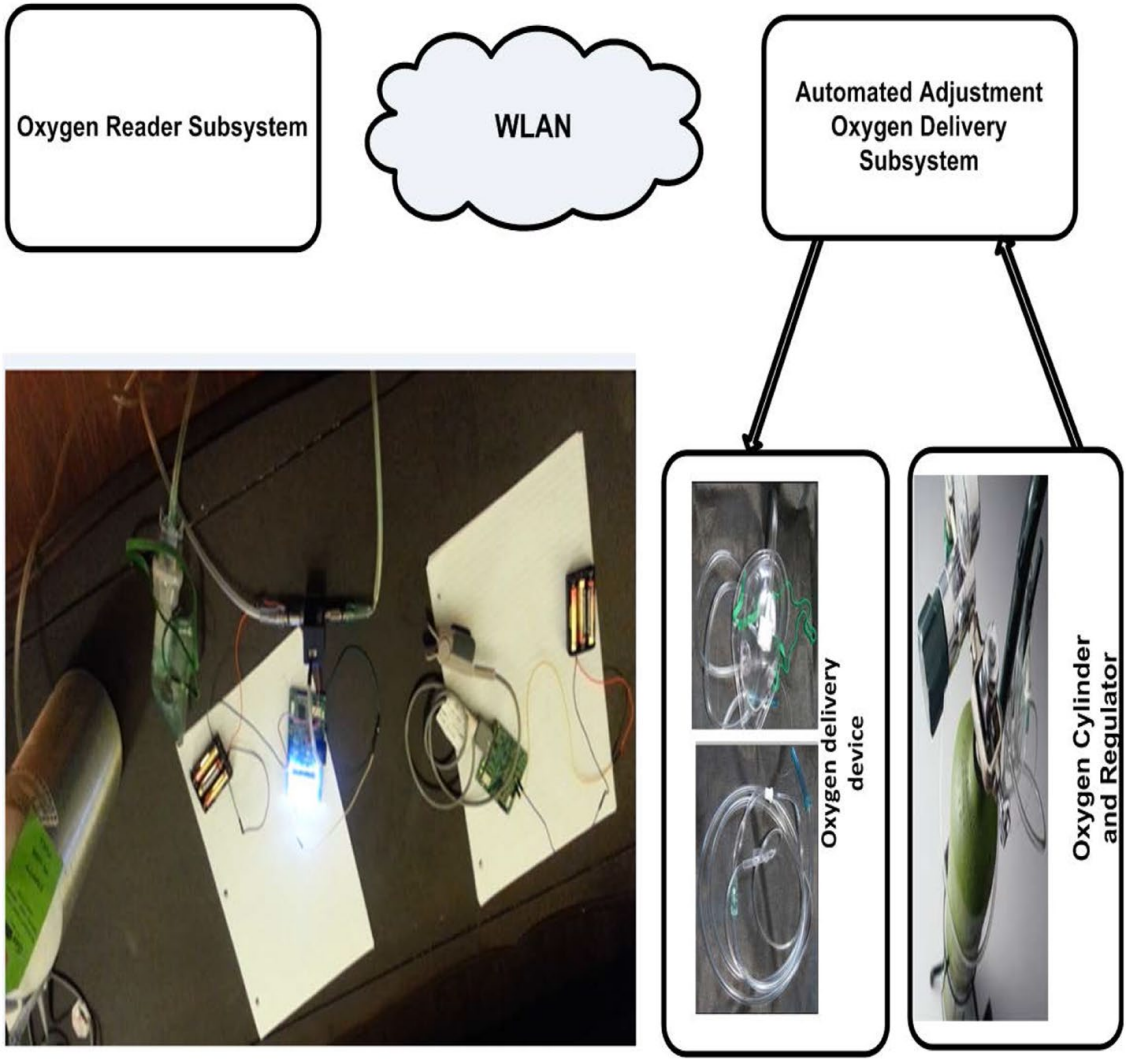
Prototype model for the Automated Oxygen Administration System

The developed prototype model for PAOAS is shown in Fig. 6. It has two subsystems that communicate wirelessly using Bluetooth technology: an Oxygen Reader Subsystem and Automated Adjustment Oxygen Delivery Subsystem. The Oxygen Reader Subsystem captures the photoplethysmography (PPG) signal non-invasively in a continuous manner using the radiation sensing technique. The captured raw measurements are processed every one second in the MCU using DSP filters, and consequently oxygen saturation level, heart rate, and perfusion index are computed and sent wirelessly using Bluetooth technology to the Automated Adjustment Oxygen Delivery Subsystem. The computed PPGs measurements are received every one second by the Automated Adjustment Oxygen Delivery Subsystem and displayed on the attached small LCD display; while the amount of supplemental oxygen (for hypoxaemic patients) is adjusted accordingly by the closed loop proportional solenoid valve.

### Description of the Oxygen Reader Subsystem

**Fig. 7:**
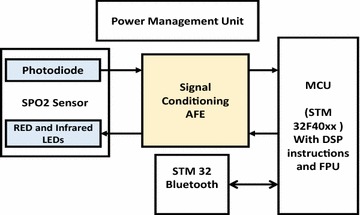
Design architecture of the Oxygen Reader Subsystem

The Oxygen Reader Subsystem consists of oxygen sensor, analog front end (AFE) unit, microcontroller, power unit, and bluetooth module as shown in Fig. 7. The selected microcontroller (STM32F40xxx) for this Subsystem has the required peripherals and capabilities to accomplish the required tasks, such as A/D, D/A, Timers, SPI, USART, DSP and power management capabilities. The microcontroller reads the raw PPG signal from the AFE circuit, where the AFE is simply a transducer circuit and a LEDs driving circuit. The transducer circuit converts the low detected current from the photodiode of the ${\text {SpO}}_{2}$ sensor to a readable voltage by the microcontroller’s A/D. The LEDs driving circuit controls switching operation and the intensities of the Red and Infra-red LEDs of the ${\text {SpO}}_{2}$ sensor based on commands from the MCU.

**Fig. 8:**
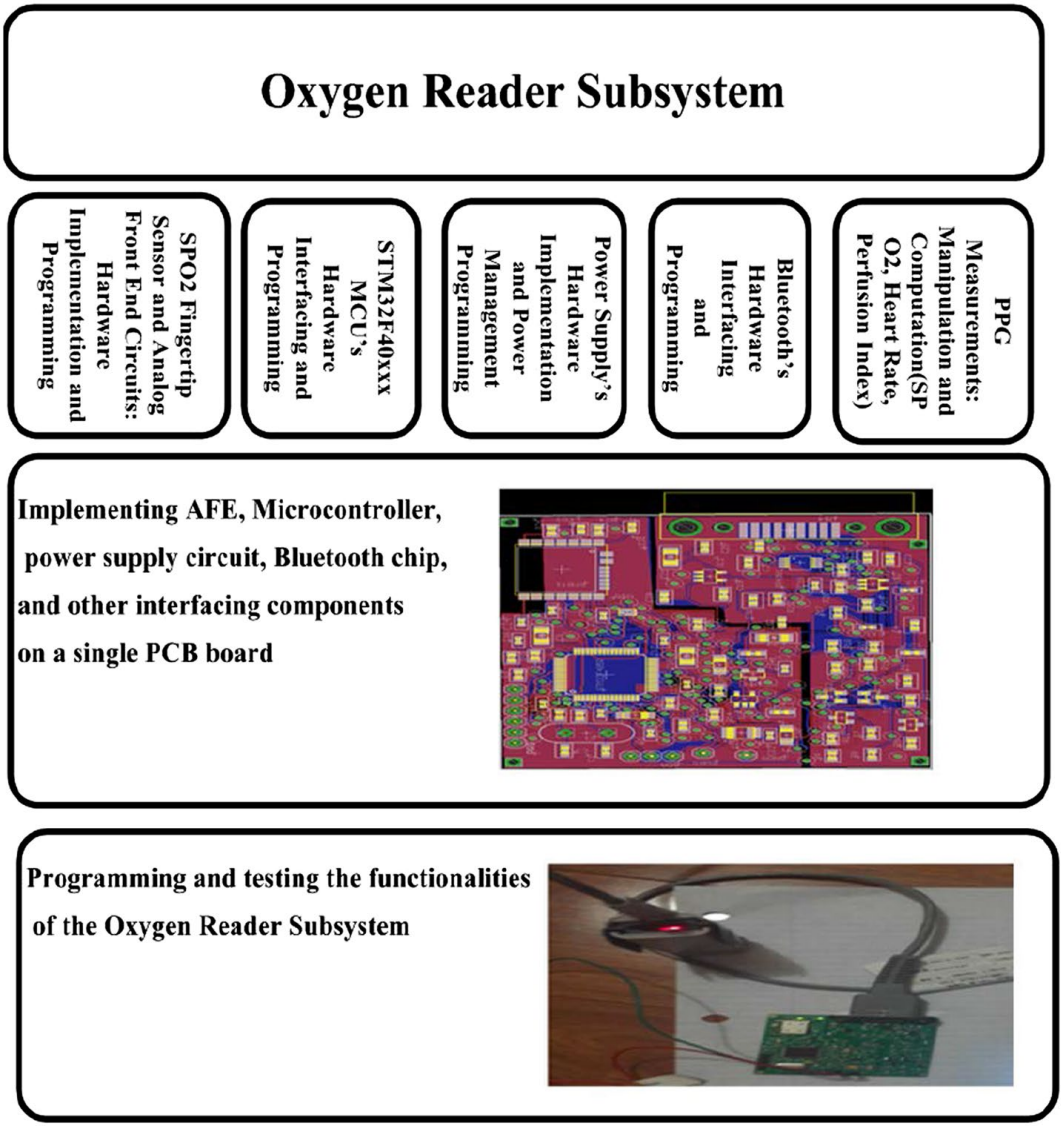
Tasks diagram for the Oxygen Reader Subsystem

The Oxygen Reader Subsystem was implemented by performing the tasks that are shown in the diagram of Fig. 8. The MCU is the main unit in the Oxygen Reader Subsystem as it has to perform and continuously control the following operations: (1) Control the LEDs switching and intensities of the ${\text {SpO}}_{2}$ sensor through the LEDs driving circuit, (2) Read the PPG signal from the ${\text {SpO}}_{2}$ sensor through the transducer circuit, (3) Manipulate the PPG signal using the DSP algorithms and compute the oxygen level and heart rate, and (4) Send wirelessly the computed oxygen level and heart rate every one second using the STM32 Bluetooth module to the Automated Adjustment Subsystem. Each subsystem was assembled on one side of a 2.14 inch $\times$ 3.19 inch two-layer PCB board. For the final product, the PCB boards for the two subsystems will be smaller as we intend to increase the number of layers and have the components assembled on both sides. The Oxygen Reader Subsystem will be a wearable earlobe, and the Automated Adjustment Oxygen Delivery System will be a handheld device that attached to the oxygen cylinder or the oxygenator.

The concept of using radiation sensing technique to compute the oxygen level in arterial blood is not new as it was first introduced in 1935 when Matthes and his colleague developed the first oxygen saturation meter that used a 2-wavelength light source with red and green lights (Matthes 1935) [later changed to Red (660 nm) and Infra-red (910 nm) lights]. The first commercialized Pulse-Oximeter was developed by Aoyagi et al. (1974) and commercialized by Biox in 1981. In 2005, Masimo company released the newest device, Co-oximeter (ex: Rad-57 handheld) to measure the oxygen saturation more accurately to overcome many limitations of traditional Pulse-Oximeter (Ruckman 2011). In this device, they used 8 different wavelengths and other algorithms to acquire and manipulate the data. Measuring the oxygen saturation level and the heart rate using radiation sensing technique is based on two basic principles: (1) The volume of blood in arteries changes with each heart beat (arterial blood is pulsatile in nature while venous blood is not), and (2) The absorption of the Hb (Deoxyhemoglobin) and ${\text {HbO}}_{2}$ (Oxyhemoglobin) for Red and Infra-red lights due to color and conformation. ${\text {HbO}}_{2}$ absorbs more Infra-red light than Hb which absorbs more Red light than ${\text {HbO}}_{2}$ (Bledsoe 2012). When a transmissive ${\text {SpO}}_{2}$ sensor is placed around a thin body part (such as an earlobe or a fingertip), part of the transmitted Red or Infra-red signal will be absorbed by the thin body part (arterial, venous, tissues, and bones) (Tamura et al. 2014); the arterial blood is pulsatile with systolic and diastolic phase of the cardiac cycle. The detected transmitted signal from the other thin body part has DC and AC components. These two components are used to compute the oxygen saturation level and the heart rate based on Beer-Lambert law that describes light attenuation through a sample of homogeneous non-scattering media or based on predefined calibration method that derived based on the experimental measurements.

**Fig. 9:**
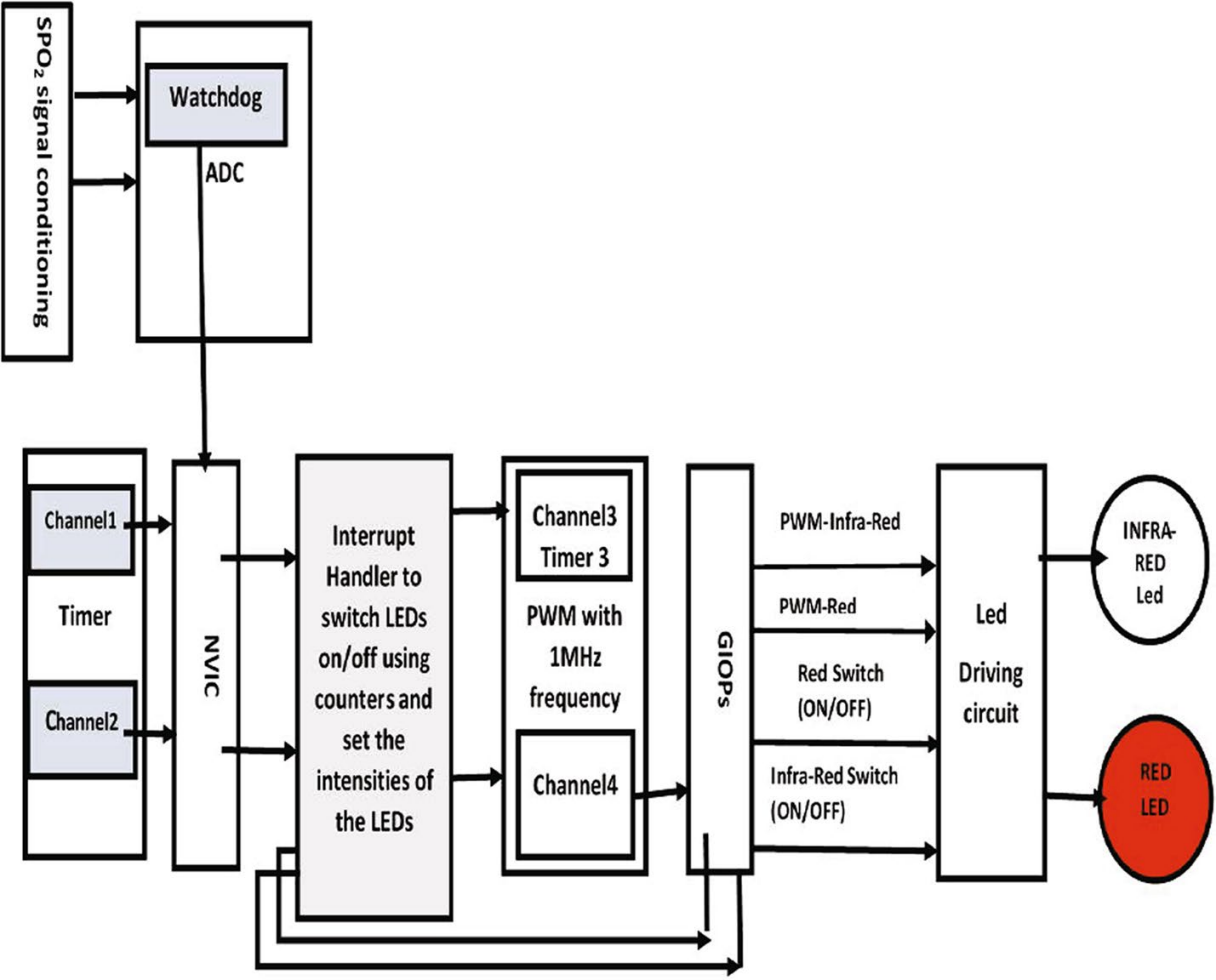
Controlling the ${\text {SpO}}_{2}$ sensors LEDs and acquiring the conditioned transmitted light measurements from the ${\text {SpO}}_{2}$ sensor

In this work, The A/D converter of the MCU captures the raw signal from ${\text {SpO}}_{2}$ sensor through the transducer circuit of the AFE with sampling rate equals to 500 samples per second. The raw measurements contain multiplexed data for the photodiode sensor for three different configurations: Red LED ON and Infra-red LED OFF, Red LED OFF and Infra-red LED OFF, and Red LED OFF and Infra-red LED ON. The LEDs driving circuit controls the LEDs switching sequences and their intensities through the output ports and PWM of the MCU as shown in Fig. 9. The MCU controls switching the LEDs in the following sequences for Red/Infra-red LEDs: ON/OFF, OFF/OFF, OFF/ON, OFF/OFF. The MCU unit de-multiplexes the captured measurements of the A/D converter to Red raw PPG signal, Infra-red raw PPG signal, and a reference signal. This means that the sampling frequency for the captured Red/Infra-red PPG signal is 125 sample/second. To reduce the power consumption when LEDs are ON, we use a timer to turn the LEDs OFF after reading the corresponding measurement for the photodiode sensor from AFE circuit. The synchronization between switching the LEDs and capturing their corresponding measurements is very important in this context to avoid capture unstable or invalid measurements.

**Fig. 10:**
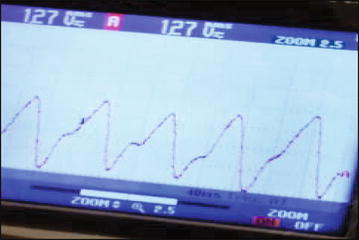
AC part of the processed PPG signal, captured and processed in the Oxygen Reader Subsystem

The raw Red PPG and Infra-red PPG signals were processed using Digital FIR and IIR filters to obtain their AC and the DC components. The filters to extract the AC and DC components of the PPG signal were: (1) Second order, low pass IIR Butterworth filter with cut-off frequency equals to 0.1 Hz for DC tracking, (2) Fourth order, high pass IIR Butterworth filter with stop frequency equals to 0.1 Hz and pass frequency equals to 0.5 Hz for DC removal, and (3) Tenth order, Low pass FIR-Window (Kaiser window with beta = 0.5) filter with cut-off frequency equals to 10 Hz to remove the noise from the AC component. The first filter was used to track the DC component, and the other two filters were used in consequence to obtain the AC component and remove the noise. These filters were designed using filter design tool in Simulink (MATLAB), then the coefficients of these filters were extracted and used with DSP library from STMicroelectronics to implement these filters and acquire the AC and DC components of the Red PPG and Infra-red PPG signals. Designing these filters was conducted while considering the frequency band for the PPG signal, which is between 0.5 and 4.0 Hz (Carr and Brown 1998; MASIMo 2001). The processed PPG signal in the Oxygen Reader Subsystem is shown in Fig. 10.

Oxygen saturation (${\text {SpO}}_{2})$ in arterial blood is defined as the ratio of the oxyhaemoglobin to the total haemoglobin as described in Eq. (3). Thus, the computation of oxygen level is based on measuring the intensity of the Red and Infra-red lights that are attenuated by a thin body part and captured by the photodiode sensor. The captured signal is divided into a DC and AC components. The DC component represents the light absorption of the tissue, venous blood, and non-pulsatile arterial blood, while the AC component represents the pulsatile arterial blood.

3 $${\text {SpO}}_{2}=\frac{{\text {HbO}}_{2}}{(Total\,Haemoglobin)} \times 100\,\%$$

The measurement of the ${\text {SpO}}_{2}$ in arterial blood is conducted by utilizing The Beer–Lambert model, where the ratio of the absorbed light for two wavelengths is computed using Eq. (4). To compute the ${\text {SpO}}_{2}$, either a lookup table is sued to match the computed R with the ${\text {SpO}}_{2}$ or a calibration equation (${\text {SpO}}_{2}=k\times R$, where k can be considered from the calibration results; Wongjan et al. 2009) is used. Clinical experimental measurements must be used in forming the calibration equation and lookup table. The ${\text {SpO}}_{2}$ in this work was computed using the empirical Eq. (5) that was formulated based on experimental measurements for the published work in Mehta et al. (2012).

4 $$R = \frac{\frac{AC_{Red}}{DC_{Red}}}{\frac{AC_{Infrared}}{DC_{Infrared}}}$$

5 $$SPO_{2} = 110-(25\times Ratio)$$

To compute the heart rate per minute, the sampling frequency of the PPG signals was multiplied by 60 s and divided by the average number of samples in each heart pulse as in Eq. (6).

6 $$HR=\frac{Sampling\, rate\times 60}{\frac{number\, of\, samples}{number\, of\, heart\, pulses}}$$

To stabilize the measurements, the weighted moving average (WMA) was used. After computing the ${\text {SpO}}_{2}$, heart rate, and perfusion index every second in the Oxygen Reader Subsystem, these measurements are sent to the STM32 Bluetooth module utilizing the USART serial connection. The Bluetooth module in turn sends these measurements wirelessly to the automated adjustment Oxygen Delivery Subsystem.

### Description of the automated adjustment Oxygen Delivery Subsystem

**Fig. 11:**
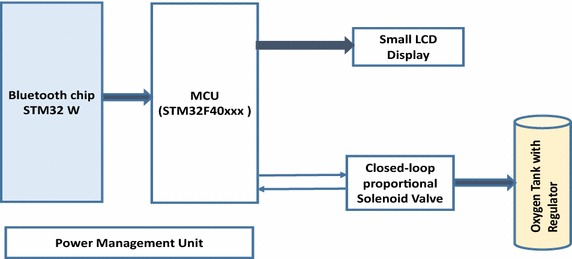
Design architecture for the Automated Adjustment Oxygen Delivery Subsystem

The hardware architecture for the Automated Adjustment Oxygen Delivery Subsystem consists of Bluetooth chip, 32-bit ARM Microcontroller, a small LCD display, power management unit,and Parker closed-loop proportional solenoid valve, as shown in Fig. 11.

**Fig. 12:**
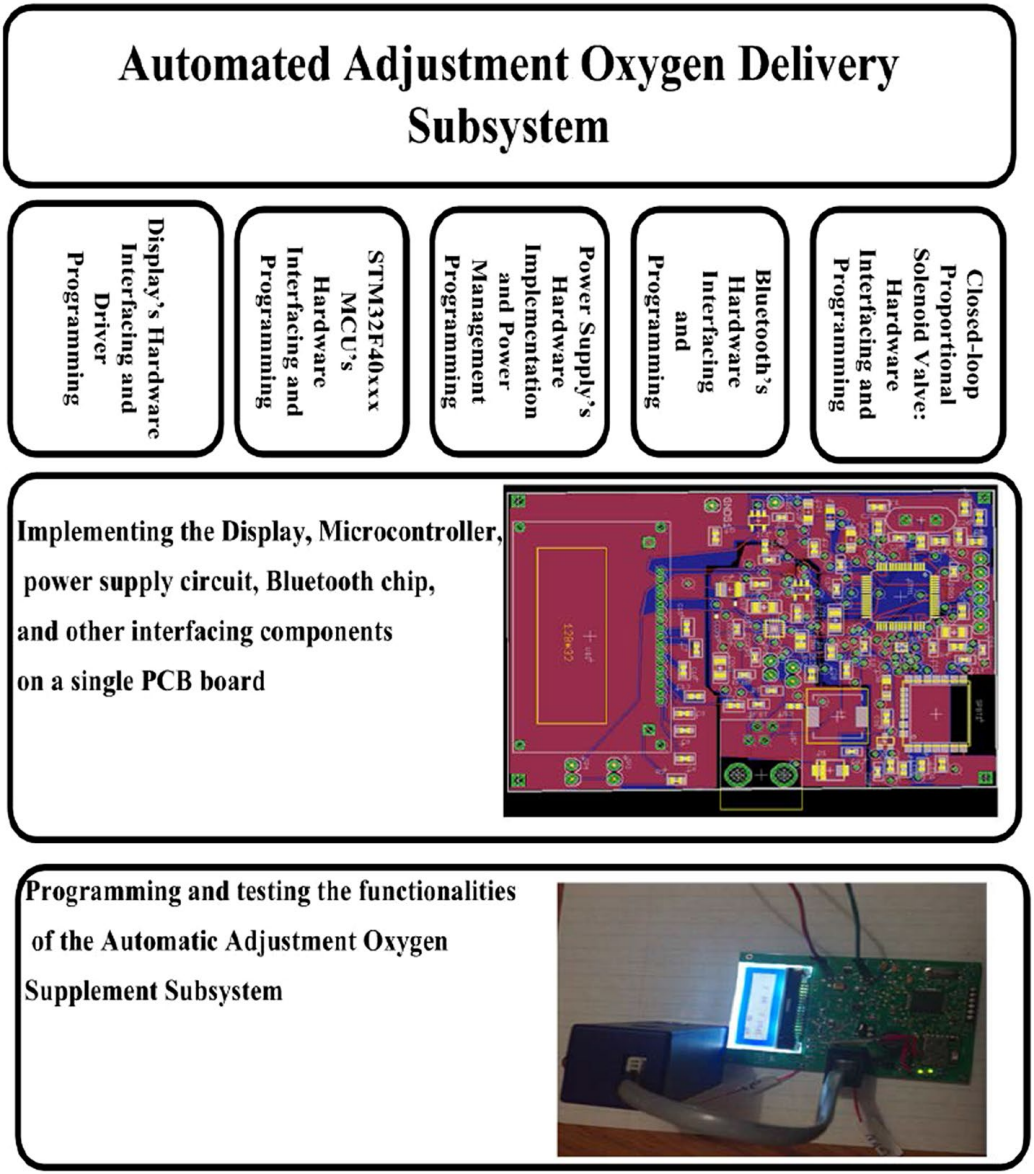
Tasks diagram for the Automated Adjustment Oxygen Delivery Subsystem

The prototype model for the Automated Adjustment Oxygen Delivery Subsystem has been implemented by performing the tasks that are shown in the Diagram of Fig. 12. In this work, we have followed the Waterfall model (McConnell 1996) where each task is divided into subtasks and integrated with the other tasks and iteratively re-visited to release the prototype model for PAOAS.

**Fig. 13:**
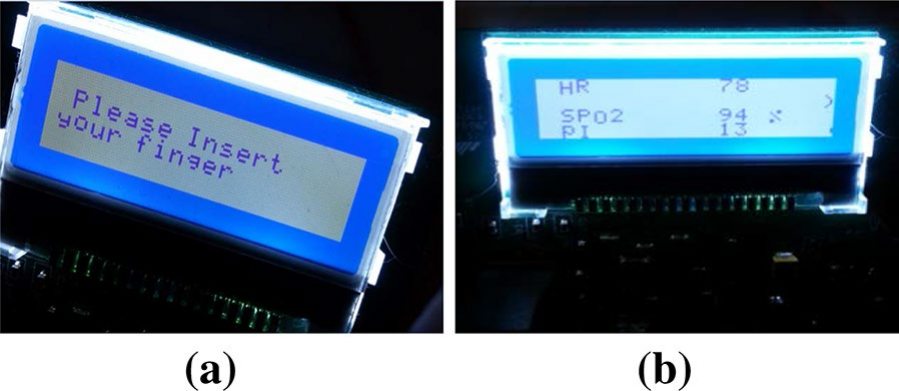
The LCD display showing **a** the message “Please Insert Your Finger”, **b** the $SpO_{2}$, heart rate, and perfusion index for the captured PPG signal

**Fig. 14:**
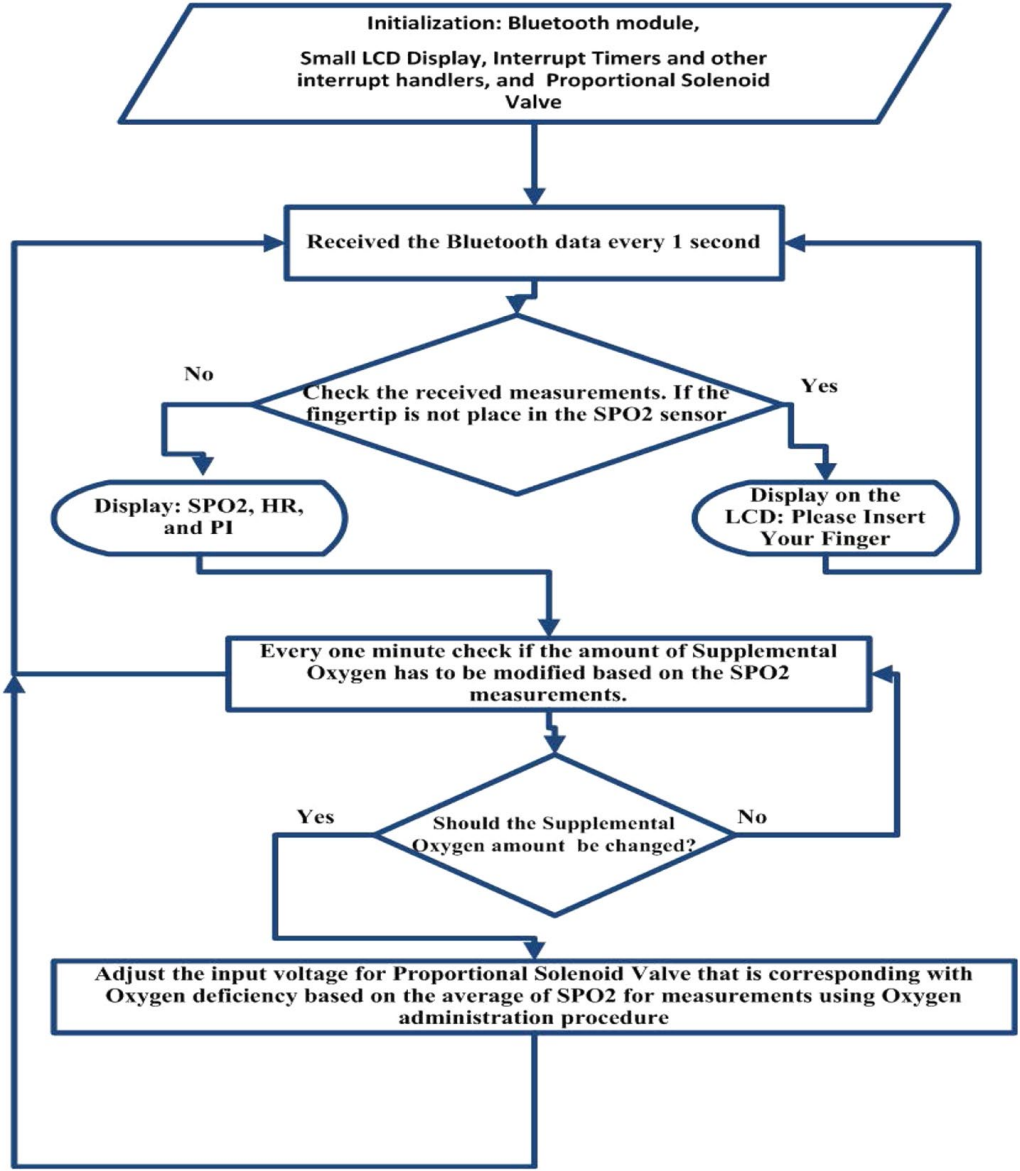
Flow chart for the Automated Adjustment Oxygen Delivery Subsystem

The Automated Adjustment Oxygen Delivery Subsystem receives data from the Oxygen Reader Subsystem through the Bluetooth module every one second. The received data is checked against predefined values. If there is no PPG readings in the received data, a message Please Insert your Finger appears in the LCD display as shown in Fig. 13a, and the proportional solenoid valve stops delivery of oxygen. If the received data carries PPG measurements, the (${\text {SPO}}_ {2}$, HR, PI) will be displayed on the LCD display as shown in Fig. 13b. The amount of oxygen that should compensate for the oxygen deficiency is determined based on the ${\text {SpO}}_{2}$ level and predefined values for oxygen administration. Consequently, the proportional solenoid valve will be controlled to allow specific defined amount of oxygen to be delivered to the hypoxaemic patient from the oxygen supplement. The closed-loop solenoid valve controls the flow rate of the delivered oxygen from the oxygen supplement (Cylinder or Oxygenator). The amount of supplemental oxygen is modified every minute to consider the ${\text {SpO}}_{2}$ level or based on other criteria for oxygen administration procedure. The Flowchart for the Automated Adjustment Oxygen Delivery Subsystem is shown in Fig. 14.

The developed prototype model for the Automated Adjustment Oxygen Delivery Subsystem is connected with Parker closed-loop proportional solenoid valve through RJ45 connector. The closed-loop proportional solenoid valve takes oxygen with pressure equals to 50 PSI as input and adjusts the amount of oxygen that is provided to the patient at its output port. The amount of oxygen is determined by the input voltage to the proportional solenoid valve from the D/A convert of the MCU that determines this amount based on the ${\text {SpO}}_{2}$ measurements and using oxygen administration algorithm. In the developed model, we used a simple algorithm to achieve a target saturation of 94–98 % for most acutely ill patients as stated in the guidelines for oxygen prescriptions guide from British Thoracic Society (BTS) (Welham et al. 2010). For the final system, we will implement the described algorithm in Fig. 1 of BTS Guidelines in Welham et al. (2010), which addresses the amount of oxygen that should be delivered to different types of patients based on their illness and needs.

### Integration and testing the Portable Oxygen Administration System

**Fig. 15:**
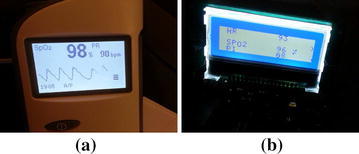
${\text {SpO}}_{2}$, HR, and PI using: **a** commercial Pulse-Oximeter, **b** prototype model for the Automated Oxygen Administration System

**Fig. 16:**
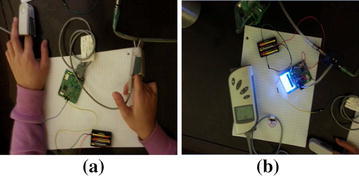
Set up for the ${\text {SpO}}_{2}$, HR, and PI using commercial Pulse-Oximeter and the prototype model for the Automated Oxygen Administration System with its two subsystems that communicate wirelessly: **a** Oxygen Reader Subsystem, **b** Automated Adjustment Oxygen Delivery Subsystem

The two subsystems of the Portable Oxygen Administration System were integrated and tested against the required functionalities. To check the capability of the developed model in measuring the oxygen saturation level and the heart rate, measurements were compared to the PPG measurements from the commercial Pulse Oximeter (H100B) by conducting experimental measurements using the set up in Fig. 16. The experiment was conducted on the same person at the same time, where her fingertip of left hand was placed in the ${\text {SpO}}_{2}$ sensor for the commercial Pulse Oximeter and the her fingertip of the right hand was placed in the ${\text {SpO}}_{2}$ sensor of the oxygen Reader Subsystem as shown in Fig. 16a. Measurements were read from both system is shown in Fig. 16b. The experiment was repeated on different people. The experiments showed very good agreement between the ${\text {SpO}}_{2}$ and heart rate measurements from our system and from the commercial Pulse-Oximeter (H100B) (see Fig. 15). The developed prototype shows good agreements for ${\text {SpO}}_{2}$ and heart rate measurements when compared to the Commercial Pulse Oximeter. The agreement was good despite the need for our system to be calibrated based on experimental measurements. During next phase of this project, we will calibrate our developed system by conducting experimental measurements on different patients at hospital settings.

### Challenges and limitations

Computer failure is a challenge that has to be addressed when designing medical systems. Computer-related failure can be defined as any event that causes a computer-based medical device to function improperly or present harm to patients or users due to failures in any of the medical systems components including software, hardware, I/O, or battery. Most computer failures are software failures (Alemzadeh et al. 2013). To address software failure in our system, we used watchdog timer mechanism to monitor if the system hangs or does not respond for a specific period of time because of any software failure. For example, if the system hangs for more than one minute, the watchdog timer will restart the system. Moreover, if there is a hardware failure, an alarm message will be displayed to notify the user.

Battery-life is another crucial factor in portable medical systems. To address the battery-life in our developed prototype model, we focused on reducing the power consumption during the design phase by considering the power consumption as a critical design parameter. This was in addition to using different power management techniques. In our final product, we will use rechargeable batteries and implement more robust power management technique to prolong the battery life.

## Summary and conclusions

In this work we investigated Oxygen therapy administration in terms of importance, limitations, and global burden of hypoxaemia. We provided a medical background about hypoxia and hypoxemia by explaining the difference between hypoxia and hypoxemia, defining the different sources causing hypoxia, classifying hypoxia into four different types based on causes, and describing how hypoxemia can be detected and measured. Furthermore, we defined the oxygen administration process, and provided the benefits of automated oxygen administration process on healthcare service and hypoxaemic patient’s life by having a PAOAS. To address the aforementioned needs and provide the hypoxaemic patient with continuous administered oxygen therapy at hospital, pre-clinic, and home settings, we introduced a prototype model for the PAOAS. The developed prototype consisted of two subsystems that communicate wirelessly using Bluetooth technology: an Oxygen Reader Subsystem, and Automated Adjustment Oxygen Delivery Subsystem. The prototype model measures the ${\text {SpO}}_{2}$ and consequently adjusts the amount of supplemental oxygen that is delivered to the patient accordingly using simple oxygen administration algorithm. The measurements for ${\text {SpO}}_{2}$, and heart rate from our system showed strong agreements with the measurements from a commercial Pulse Oximeter. We expect to achieve higher reading accuracy after calibrating our system based on experimental measurements. Our final product will be small, the Oxygen Reader Subsystem will be wearable earlobe device and the Automated Adjustment Oxygen Delivery Subsystem will be more intelligent by implementing more robust oxygen administration process that could help in accelerating the weaning process for patient who needs oxygen therapy for short-term.

The PAOAS developed in this work used Bluetooth technology for transferring data between its two subsystems, thus patient’s measurements can be sent to a patient’s cell phone using Bluetooth technology for further transfer to healthcare providers for further monitoring and administration. By implementing an intelligent algorithm for oxygen weaning process, we expect our system will help to accelerate the oxygen weaning process for patients who need oxygen therapy for a short term.
